# Academic Productivity and Artificial Intelligence: Beyond Time Constraints

**DOI:** 10.31662/jmaj.2025-0289

**Published:** 2025-09-05

**Authors:** Shigeki Matsubara

**Affiliations:** 1 Department of Obstetrics and Gynecology, Jichi Medical University, Tochigi, Japan; 2 Department of Obstetrics and Gynecology, Koga Red Cross Hospital, Koga, Japan; 3 Medical Examination Center, Ibaraki Western Medical Center, Chikusei, Japan

**Keywords:** artificial intelligence, motivation, paper, paper productivity, research

## Dear Editors,

Kaneda and Ozaki ^(1)^ hit the point. There have been many studies on artificial intelligence (AI) that discussed its use in medicine in general, without distinguishing between its use in research and/or writing and its use in medical activities/services ^(2)^. By making this distinction, they claimed, let’s ensure “research time” by streamlining doctors’ administrative jobs. I state my view regarding “research time” and “academic productivity.”

Kaneda and Ozaki are concerned about Japanese doctors’ academic productivity: limited research time accounts for it. Indeed, research requires time: I had no publication during my university hospital ward chief era (1992-1996). I participated in almost all emergent obstetric surgeries in a 24/7 manner, along with heavy administrative jobs. Although I love writing and eventually wrote 620 PubMed-indexed papers, this was a “blank period” in terms of paper production.

Data showed that protected research time enhanced paper productivity ^(3)^ and engaging younger doctors in research led to continued paper productivity ^(4)^. Providing time for research, especially for younger-generation doctors, may enhance academic productivity.

I believe a fundamental factor other than “time” exists to enhance academic activity, especially among younger generations. When I was a medical student, we witnessed laboratory lights shining at midnight: doctor-researchers looked lively. We, medical students, have strong respect for our seniors’ struggling efforts. Professors told their own research stories merrily. Although I didn’t learn how to research, bathing in that atmosphere led me to an academic career. The “yearning” for research planted then became a strong lifelong motivation to continue research.

Although there are no objective data, the current atmosphere in the medical world looks to me like the following. “Do not talk about your old stories, about 24/7 work or laboratory’s midnight lights.” I agree. The “harder, the better” era has ended. We, older generations, should not speak as if the past era were wonderful. However, seasoned doctor-researchers should openly voice, “Research and writing are something yearned for.” “No time, no research” is true. “No yearning, no research” is also true ^(5)^.

I believe that we must not merely protect younger doctors from burnout, but more positively secure their mental and physical reserve for research. AI should be used to streamline doctors’ administrative jobs, and work-style reform should also be enhanced, both under the condition of patients’ safety. Let’s extinguish “blank time” for ambitious younger doctors.

Here, whether AI streamlines research and paper writing is another matter. More importantly, whether it preserves the “yearning” for research and writing remains a question deserving careful consideration.

## Article Information

### Acknowledgments

The author thanks Daisuke Matsubara (Division of Community and Family Medicine and Department of Pediatrics, Jichi Medical University, Japan) for his help.

### Author Contributions

Shigeki Matsubara: Identification of the significance and Manuscript writing.

### Conflicts of Interest

None

### Approval of Institutional Review Board

Not applicable.

### Patient Anonymity

Not applicable.

### Informed Consent

Not applicable.

### Data Availability

Data sharing is not applicable to this article, as no new data were created or analyzed in this study.
